# Sun Protection Behaviors Associated with Self-Efficacy, Susceptibility, and Awareness among Uninsured Primary Care Patients Utilizing a Free Clinic

**DOI:** 10.1155/2015/753681

**Published:** 2015-09-06

**Authors:** Akiko Kamimura, Maziar M. Nourian, Jeanie Ashby, Ha Ngoc Trinh, Jennifer Tabler, Nushean Assasnik, Bethany K. H. Lewis

**Affiliations:** ^1^Department of Sociology, University of Utah, Salt Lake City, UT 84112, USA; ^2^School of Medicine, University of Utah, Salt Lake City, UT 84132, USA; ^3^Maliheh Free Clinic, Salt Lake City, UT 84107, USA; ^4^Department of Sociology, Vietnam National University, Hanoi, Vietnam; ^5^Health Society and Policy, University of Utah, Salt Lake City, UT 84112, USA; ^6^Department of Dermatology, University of Utah, Salt Lake City, UT 84132, USA

## Abstract

*Background*. Skin cancer is the most commonly diagnosed form of cancer in the United States (US). However, knowledge, behaviors, and attitudes regarding sun protection vary among the general population. The purpose of this study is to examine sun protection behaviors of low-income primary care patients and assess the association between these health behaviors and the self-efficacy, susceptibility, and skin cancer awareness. *Methods*. Uninsured primary care patients utilizing a free clinic (*N* = 551) completed a self-administered survey in May and June 2015. *Results*. Using sunscreen was the least common tactic among the participants of this study. Skin cancer awareness and self-efficacy are important to improve sun protection behaviors. Spanish speakers may have lower levels of skin care awareness compared to US born and non-US born English speakers. Male and female participants use different sun protection methods. *Conclusion*. It is important to increase skin cancer awareness with self-efficacy interventions as well as education on low-cost sun protection methods. Spanish speaking patients would be a target population for promoting awareness. Male and female patients would need separate gender-specific sun protection education. Future studies should implement educational programs and assess the effectiveness of the programs to further promote skin cancer prevention among underserved populations.

## 1. Introduction

Skin cancer is the most commonly diagnosed form of cancer in the United States (US) [1]. The incident rate of cancer is known to vary by race and ethnicity: non-Hispanic whites are most susceptible to skin cancer (25 per 100,000), followed by Hispanics (4 per 100,000), while blacks are least likely to be diagnosed with skin cancer (1 per 100,000) [1]. Yet, racial or ethnic minority groups tend to have higher mortality and morbidity risks from skin cancer compared to majority groups [2]. Decreasing unprotected ultraviolet exposure is effective in preventing skin cancer [3]; thus, wearing sunscreen and wearing sun protective clothing are common recommendations for sun protection [4]. However, knowledge, behaviors, and attitudes regarding sun protection vary among the general population [5].

Variation in the knowledge, behaviors, and attitudes toward sun protection may be related to group differences in self-efficacy, perceived susceptibility, and skin cancer awareness; it is well established that individual level self-efficacy, perceived susceptibility, and awareness are important constructs influencing health behaviors [6], including sun protection behaviors and beliefs. Self-efficacy refers to confidence in one's ability to change behaviors [7] and affects sun protection intention and behavior [8–10]. Higher levels of self-efficacy are related to higher levels of desire to improve sun protection [11]. In addition, one's belief about the chance to get a disease, that is, perceived “susceptibility,” is related to higher levels of motivation for sun protection behaviors [12]. Higher levels of perceived susceptibility are associated with higher levels of desire to get assistance to improve sun protection [11]. However, perceived susceptibility of skin cancer is low in general [13]. Awareness is an application for a cue to action to change health behaviors [6]. Yet skin cancer awareness is low overall, especially among minority patients [14].

Sociodemographic risk factors for reduced practice of sun protection include younger age, male gender, non-Hispanic white, Hispanic ethnicity, and low education level [5, 15, 16]. While previous studies have focused on some of the sociodemographically high risk groups, few studies examined sun protection behaviors among low-income primary care patients. In general, primary care patients are less likely to receive skin cancer screening compared to screening of other cancers [17]. Low-income primary care patients may be particularly at risk for poor skin cancer awareness and use of sun protection due to the limited access to resources. Thus, the purpose of this study is to examine sun protection behaviors of low-income primary care patients and assess the association between these health behaviors and the self-efficacy, susceptibility, and skin cancer awareness. This study increases knowledge about sun protection behaviors and influencing factors in order to develop effective intervention strategies to promote skin cancer prevention among low-income patients in a primary care setting.

In particular, this study focuses on low-income and uninsured patients who are utilizing a free clinic providing primary care for the underserved. Free clinics provide free or reduced fee healthcare to individuals who lack access to primary care and are socioeconomically disadvantaged in the US [18, 19]. Most free clinics rely on volunteer providers and staff and limited financial resources [20]. Approximately 40% of free clinic patients are immigrants [21]. Patients who utilize free clinics suffer from a wide variety of medical conditions such as respiratory diseases, circulatory diseases, and mental disorders, [22] and tend to experience poor physical and mental health and low levels of health-related quality of life [23, 24]. To the best of our knowledge, there are no previous studies that examine skin cancer risk, sun protection, and related dermatological health issues of free clinic patients.

## 2. Methods

### 2.1. Overview

The current community-based research project was conducted at a free clinic in the Intermountain West. The clinic staff collaborated with this research team to develop the survey instrument, study protocol, participant recruitment strategies, and interpretation of study results. The clinic provides free healthcare services, mostly routine health maintenance and preventative care, for uninsured individuals who live below the 150th percentile federal poverty level and do not have access to employer-provided or government-funded health insurance. The primary focus of the clinic is routine health maintenance and preventative care for chronic conditions such as diabetes and cardiovascular diseases. The clinic is staffed by six full-time paid personnel and over 300 active volunteers, including approximately 60 volunteer interpreters. The clinic, which has been in operation since 2005, has no affiliation with religious organizations and is funded by nongovernmental grants and donations. The clinic is open five days a week. The number of patient visits was 18,967 in 2013. The clinic does not ask patients to provide documentation of legal residency or citizenship and serves undocumented immigrants as well as US citizens and documented immigrants.

### 2.2. Study Participants and Data Collection

Participants were aged 18 years or older, spoke and read English or Spanish, and were patients of the clinic. A bilingual translator translated English materials into Spanish. Another bilingual translator conducted back-translation from Spanish to English. The third bilingual translator checked accuracy of the translation. Participants were divided into three groups, namely, US born English speakers, non-US born English speakers, and Spanish speakers, because previous studies on free clinic patients have indicated that these three populations have different sociodemographic characteristics, physical and mental health status, and healthcare needs [23–25].

Prior to data collection, the Institutional Review Board (IRB) approved this study. The data were collected for two months, in May and June 2015. Recruitment occurred at the free clinic by distributing flyers to patients in the waiting room. If a potential participant expressed interest in participating in the study, he or she received a consent cover letter and a self-administered paper and pencil survey. Members of the study team were available to answer any questions while participants were taking the survey. Participants received sample sunscreen or hand sanitizer (US $1 or less value) at the completion of the survey. The research assistants who collected surveys checked item nonresponses immediately after submission and asked the participant to fill in missing parts if there were any, whenever it was possible. Only completed surveys were included in the analysis.

### 2.3. Measures

#### 2.3.1. Sun Protection Behaviors

Five sun protection related questions were extracted from the Health Information National Trends Survey, the National Cancer Institute (http://hints.cancer.gov/default.aspx): (1) “When you go outside for more than 1 hour on a warm sunny day, how often do you wear long pants?”; (2) “When you go outside for more than 1 hour on a warm sunny day, how often do you wear sunscreen?”; (3) “How often do you stay in the shade or under an umbrella?”; (4) “How often do you wear a hat?”; and (5) “How often do you wear a shirt with sleeves that cover your shoulders?” The questionnaire uses a five-point Likert scale (0 = never to 4 = always) to determine the levels of sun protection behaviors.

#### 2.3.2. Self-Efficacy

Self-efficacy was measured by the General Self-Efficacy Scale [26]. The scale has 10 items and uses a 4-point Likert scale (1 = not at all true, 4 = exactly true). The examples of the items include “I can always manage to solve difficult problems if I try hard enough” and “I can usually handle whatever comes my way.” The scoring is based on a sum of all items (score range: 0–40). While there is no specific cutoff point to identify high or low levels of self-efficacy, the mean score of US-American adults is 29.48 [27]. This scale has been used in many countries and languages and its validity and reliability have been tested [27]. Cronbach's alpha for this study population was 0.905.

#### 2.3.3. Skin Cancer Susceptibility and Awareness

Skin cancer susceptibility and awareness were measured by the scale based on the Health Belief Model [28]. There are six items for the susceptibility subscale (e.g., “My chances for getting skin cancer are high”) and six items for the awareness subscale (e.g., “It's important for me to wear a hat when outside in the sun”). The mean score for each subscale was used for analysis. The scale uses a 5-point Likert scale (5 = strongly agree, 1 = strongly disagree). Higher scores indicate higher levels of perceived susceptibility or awareness. Cronbach's alpha for this study population was 0.825 for the susceptibility subscale and 0.921 for the awareness subscale.

#### 2.3.4. Demographic Information

Demographic questions included age, gender, race/ethnicity, education level, employment status, marital status, US born or not, country of origin, length of years living in the US (non-US born participants only), and length of years as a patient of the free clinic (2+ years or less).

### 2.4. Data Analysis

Data were analyzed using SPSS (version 22). The participants were divided into three groups for comparison: US born English speakers, non-US born English speakers, and Spanish speakers. Descriptive statistics were used to capture the distribution of the outcome and independent variables. The three groups of the participants were compared using Pearson's Chi-square tests for categorical variables and analysis of variance (ANOVA) for continuous variables. General linear model multivariate regression analysis was conducted to assess predictors of sun protection behaviors, including self-efficacy, susceptibility, awareness, and sociodemographic factors. Sociodemographic factors for the regression analysis were selected based on previous studies on free clinic populations [23, 24].

## 3. Results

Table 1 describes sociodemographic characteristics of 551 participants of a convenience sample (164 US born English speakers, 129 non-US born English speakers, and 258 Spanish speakers) and descriptive statistics of sun protection behaviors, self-efficacy, susceptibility, and awareness. Based on the average number of patient visits per day (75 visits/day) and the duration of the survey collection (40 days), the estimated participation rate was 58.8%. The average age of the participants was 44.37 (SD = 13.66). More than 65% of the participants were women (*n* = 365, 66.2%). Approximately 60% of the participants (*n* = 343, 62.3%) self-identified as Hispanic, Latino, or Latina. One-quarter of the participants (*n* = 138, 25%) were white. Nearly 45% of the participants (*n* = 243, 44.1%) reported having some college or higher levels of education. US born English speakers had much higher percentage of those with some college or higher levels of education (*n* = 103, 62.8%) than non-US born English speakers (*n* = 64, 49.6%) and Spanish speakers (*n* = 76, 29.5%) (*p* < 0.01). Likewise approximately 45% of the participants (*n* = 244, 44.3%) were currently employed. Non-US born English speakers (*n* = 73, 56.6%) and Spanish speakers (*n* = 147, 57%) were more likely to be married compared to US born English speakers (*p* < 0.01). One-third of the participants (*n* = 176, 31.9%) were US born. Among those who are not US born, the average length of living in the US was 16.2 (SD = 11.92) among non-US born English speakers and 13.98 (SD = 8.1) among Spanish speakers. The participants were from 40 countries (not shown in the table). Besides the US, Mexico had the largest number of participants (*n* = 192, 34.8%) followed by Tonga (*n* = 19, 3.4%) and Peru (*n* = 18, 3.3%). Half of the participants (*n* = 284, 51.5%) had been a patient of the free clinic for two years or longer.

The common sun protection behaviors among all groups were wearing long pants (mean = 2.70, SD = 1.17) and shirts with sleeves (mean = 2.74, SD = 1.20). Using sunscreen was the least common (mean = 1.33, SD = 1.28). Spanish speakers were more likely to wear a hat but were less likely to wear shirts with sleeves compared to US born and non-US born English speakers. The overall levels of self-efficacy were very similar to that among the US general population [27]. Spanish speakers had the highest levels of self-efficacy. Spanish speakers also reported higher levels of susceptibility but lower levels of awareness than US born and non-US born English speakers.

Table 2 presents the predictors of sun protection behavior. Higher levels of self-efficacy were associated with higher levels of sunscreen use. Higher levels of awareness were associated with higher levels of using sunscreen and shade/umbrella and of wearing a hat and shirts with sleeves. Female participants were more likely to use sunscreen and shade/umbrella but were less likely to wear a hat and shirts with sleeves compared to male participants. Lastly, Spanish speakers were less likely to wear a hat compared to the other two groups.

## 4. Discussion

This study examined sun protection behaviors associated with self-efficacy, perceived susceptibility, and awareness among low-income primary care patients utilizing a free clinic for the uninsured. While using sunscreen is the common sun protection practice compared to wearing a hat or sleeves and/or using a shade among the general US public [29], using sunscreen was the least common tactic among the participants of this study. These results suggest that low-income primary care patients tend to use different sun protection methods from the general public. This study has three main findings which may provide useful information for improving or facilitating sun protection behaviors among this population. First, skin cancer awareness and self-efficacy are important to improve sun protection behaviors. Second, Spanish speakers may have lower levels of skin care awareness compared to US born and non-US born English speakers. Third, male and female participants use different sun protection methods.

The results of this study suggest that awareness and self-efficacy, especially awareness, are important to promote sun protection behavior among low-income primary care patients. The mean score of skin cancer awareness among this study population (2.75) was lower than that of general public (3.6) [28]. Promoting skin cancer awareness may not necessarily be the main focus for free clinics given that free clinics tend to target treating patients' chronic conditions such as diabetes, hypertension, and heart disease [22]. However, because skin cancer is a common cancer, sun protection needs to be promoted among low-income patients utilizing a free clinic. Since free clinics are mostly volunteer-based [21], it may be challenging for volunteer physicians to provide skin cancer interventions. Using nonphysician providers potentially increases skin cancer screening [30] and may be feasible for free clinics to implement skin cancer prevention education. Since individuals who received self-efficacy interventions are more likely to accept sun protection messages than those without interventions [31], self-efficacy interventions should be included in skin cancer prevention education to make the educational programs effective. At the same time, the low use of sunscreens among the participants may be clearly associated with the cost of these products. Thus, interventions in skin awareness or self-efficacy may not achieve positive results because of cost of sunscreens. In this scenario other measures of sun protection such as clothes and hats may be more efficient.

While previous studies suggest that Hispanics have low levels of skin cancer susceptibility [32], the results of the current study indicate that Spanish speakers may be at greater risk for skin cancer given that Spanish speakers reported lower levels of skin cancer awareness compared to US born or non-US born English speakers. There are some barriers to engaging sun protection behaviors; for example, wearing sunscreen or protective clothing is “not part of my daily routine” was a common statement in a study analyzing barriers to sun protection in Hispanics [33]. Given this, sun protection behaviors practiced by Hispanics need to be improved [34]. However, individuals of Hispanic origin are not homogeneous and there are differences in sun protection behaviors across Hispanic subgroups: for example, Mexican heritage Hispanics are more likely to perform sun protection behavior but also experience more sunburns more often compared to other Hispanic subgroups [15]. It is therefore important to develop sun protection educational programs for Spanish speaking patients that consider within group differences in sun protection behaviors.

The results of this study also indicate that female participants are more likely to use sunscreen and shade/umbrella but are less likely to wear hat and shirts with sleeves for sun protection compared to men. Previous studies show that men are less likely to practice sun protection than women [5, 35]. Based on the results of this study, men and women use different methods for sun protection. Gender differences in clothing preference or the use of lotion may affect the choice of sun protection methods. Gender-specific sun protection interventions are necessary so that male and female patients can choose the methods with which they are familiar and may potentially implement.

This study has limitations. The cross-sectional design of this study examines associations but does not assess causal relationships among variables. Patients who were not literate in either English or Spanish were not included in this study. Non-US born English speakers have very diverse backgrounds but did not have a sample size large enough to divide them into subgroups. While participants were not selected based on specific skin types, there is a possibility that the high percentage of the minority groups (Hispanic or Asian/Pacific Islander) could affect the results of this study, which were very different from the US national average. In addition, the participants were not asked about their reasons for visiting the clinic or their health status. Because the clinic provides services primarily for chronic or comorbid conditions such as diabetes and cardiovascular diseases, patient health status could have affected the results of low interest in sun protection. While this study is based on one free clinic, the results of this study can be valuable to other free clinic populations and increase knowledge about free clinic patients who are significantly understudied because the free clinic that carried out this study shares common characteristics with other free clinics such as uninsured patients only, income requirements, 0% revenue from government, no affiliation with other organizations (an independent organization), and volunteer providers [21]. The low-income primary care patient population has not been well studied in sun protection behaviors before. This study provides new knowledge about sun protection and skin cancer prevention among the population who have been understudied.

## 5. Conclusion

Sun protection education and skin cancer prevention strategies among low-income primary care patients have not been well examined. This study contributes to the increased knowledge about the understudied population related to sun protection behaviors and skin cancer preventions. It is important to increase skin cancer awareness with self-efficacy interventions as well as education on low-cost sun protection methods. Spanish speaking patients would be a target population for promoting awareness. Male and female patients would need separate gender-specific sun protection education. Future studies should implement educational programs and assess the effectiveness of the programs to further promote skin cancer prevention among underserved populations.

## Figures and Tables

**Table 1:** Sociodemographic characteristics of participants.

	Total (*N* = 551)	US born English speakers (*n* = 164)	Non-US born English speakers (*n* = 129)	Spanish speakers (*n* = 258)	*p* value^a^
Mean age, years	44.37 (13.66)	43.07 (14.19)	43.90 (16.38)	45.46 (11.64)	N.S.
Female	365 (66.2)	103 (62.8)	78 (60.5)	184 (71.3)	N.S.
Race/ethnicity					
White	138 (25.0)	115 (70.1)	12 (9.3)	10 (3.9)	<0.01
Hispanic/Latino/Latina	343 (62.3)	33 (20.1)	62 (48.1)	248 (96.1)	<0.01
Asian or Pacific Islander	49 (8.9)	5 (3.0)	44 (34.1)	0	
Some college or higher	243 (44.1)	103 (62.8)	64 (49.6)	76 (29.5)	<0.01
Currently employed	244 (44.3)	68 (41.5)	54 (41.9)	122 (47.3)	N.S.
Currently married	252 (45.7)	32 (19.5)	73 (56.6)	147 (57.0)	<0.01
US born	176 (31.9)	164 (100)	0	12 (4.7)	
Years in the US (non-US born only)			16.20 (11.92)	13.98 (8.10)	
Patient of the clinic (2 years or longer)	284 (51.5)	103 (62.8)	71 (55.0)	110 (42.6)	<0.01
Sun protection behavior					
Long pants	2.70 (1.17)	2.62 (1.21)	2.57 (1.14)	2.81 (1.14)	N.S.
Sunscreen	1.33 (1.28)	1.40 (1.21)	1.29 (1.36)	1.30 (1.29)	N.S.
Shade or umbrella	1.95 (1.14)	1.97 (1.13)	1.96 (1.23)	1.93 (1.11)	N.S.
Hat	1.51 (1.34)	1.36 (1.30)	1.39 (1.32)	1.66 (1.36)	<0.05
Shirts with sleeves	2.74 (1.20)	2.93 (1.15)	2.84 (1.22)	2.56 (1.19)	<0.01
Self-efficacy	30.85 (5.64)	31.03 (5.07)	29.48 (5.59)	31.48 (5.94)	<0.01
Skin cancer susceptibility	2.72 (0.90)	2.73 (0.82)	2.20 (0.82)	3.00 (0.88)	<0.01
Skin cancer awareness	2.75 (1.16)	3.45 (0.76)	3.46 (0.96)	1.88 (0.87)	<0.01

Number (%) or mean (SD). N.S.: not significant.

^a^ *p* value denotes significance from Pearson's Chi-square tests between categorical variables (for cell size ≥5 only) and ANOVA tests for continuous variables comparing US born English speakers, non-US born English speakers, and Spanish speakers.

**Table 2:** Predictors of sun protection behavior (*N* = 551).

Dependent variables	Long pants *β*	*p* value	Sunscreen *β*	*p* value	Shade/umbrella *β*	*p* value	Hat *β*	*p* value	Sleeves *β*	*p* value
Independent variables										
Age	0.01	N.S.	−0.003	N.S.	0.01	<0.05	0.02	<0.01	0.004	N.S.
Female	0.01	N.S.	0.58	<0.01	0.29	<0.05	−0.48	<0.01	−0.26	<0.05
Some college or higher	−0.15	N.S.	0.03	N.S.	0.06	N.S.	0.02	N.S.	−0.07	N.S.
Employed	0.07	N.S.	−0.02	N.S.	−0.12	N.S.	−0.07	N.S.	0.09	N.S.
Married	0.15	N.S.	0.15	N.S.	0.05	N.S.	0.16	N.S.	0.15	N.S.
Clinic patient, 2+ years	0.03	N.S.	−0.07	N.S.	0.13	N.S.	−0.002	N.S.	0.16	N.S.
Self-efficacy	−0.003	N.S.	0.03	<0.05	0.00	N.S.	0.02	N.S.	−0.004	N.S.
Susceptibility	0.03	N.S.	0.03	N.S.	−0.10	N.S.	−0.03	N.S.	0.00	N.S.
Awareness	0.04	N.S.	0.25	<0.01	0.22	<0.01	0.24	<0.01	0.16	<0.05
US born English speakers^#^	−0.09	N.S.	−0.16	N.S.	−0.30	N.S.	−0.70	<0.01	0.11	N.S.
Non-US born English speakers^#^	−0.22	N.S.	−0.31	N.S.	−0.37	N.S.	−0.72	<0.01	−0.07	N.S.
(Intercept)	2.33	<0.01	−0.45	N.S.	1.10	<0.05	0.25	N.S.	2.22	<0.01

Multivariate tests										
Effect size	0.02									
*F*	1.85									
*p* value	<0.05									

Multivariate regression analysis (general linear model). *p* values are based on parameter estimates. N.S.: not significant.

Multivariate tests based on Wilks' lambda.

^#^Reference variable (reference = Spanish speakers).
